# The crowded crossroad to angiogenesis in systemic sclerosis: where is the key to the problem?

**DOI:** 10.1186/s13075-016-0937-x

**Published:** 2016-02-05

**Authors:** Mirko Manetti, Serena Guiducci, Marco Matucci-Cerinic

**Affiliations:** Department of Experimental and Clinical Medicine, Section of Anatomy and Histology, University of Florence, 50134 Florence, Italy; Department of Experimental and Clinical Medicine, Division of Rheumatology, AOUC, University of Florence, 50139 Florence, Italy

## Abstract

In systemic sclerosis (SSc), peripheral vasculopathy is characterized by a progressive and irreversible loss of capillaries following endothelial cell injury, due to defects in both vascular repair and expected increase in new vessel growth (angiogenesis). The discovery of key molecular targets may help to develop the most effective therapeutic strategy for the SSc-related vasculopathy. A pathway worth targeting in SSc may include vascular endothelial growth factor, 165b isoform, an endogenous angiogenesis inhibitor abnormally expressed and released by different cell types, including activated endothelial cells and platelets.

Dysregulated expression of several proangiogenic and antiangiogenic factors has been implicated in the dysfunction of angiogenesis in systemic sclerosis (SSc). Into this complex scenario comes the study recently published in *Arthritis Research & Therapy* by Hirigoyen et al. [1], who report new data implicating an inhibitory splice variant of vascular endothelial growth factor (VEGF)-A, namely VEGF_165_b, in the inhibition of angiogenesis by platelets in SSc. These findings are part of an intriguing chain of data as several studies have shown that VEGF-A, one of the most potent promoters of angiogenesis, is markedly increased in SSc skin and circulation despite clear evidence of an insufficient angiogenic response [2, 3]. This was considered the “SSc paradox” until it became evident recently that early studies could not distinguish between proangiogenic VEGF_165_ and antiangiogenic VEGF_165_b isoforms generated by alternative splicing in VEGF-A pre-mRNA [4]. In most pathologic angiogenic conditions, such as cancers, VEGF_165_ predominates. On the contrary, this crucial balance between isoforms shifts in favor of the expression of VEGF_165_b in SSc (Fig. 1) [4, 5]. VEGF_165_b appears selectively overexpressed in different cell types of SSc dermis, including endothelial cells (ECs), fibroblasts, and perivascular mononuclear inflammatory cells [5]. In vitro studies further revealed that SSc dermal microvascular endothelial cells (MVECs) express and release elevated levels of VEGF_165_b [5]. Moreover, the increased VEGF_165_b plasma levels in SSc correlate with the extent of nailfold capillary architectural derangement and loss, suggesting their potential utility as a biomarker reflecting disease severity [6]. In this context, the study by Hirigoyen et al. [1] adds new valuable information showing that, in SSc, platelets store high levels of VEGF_165_b. It could therefore be suggested that platelets may be a major source of circulating VEGF_165_b in SSc, in particular following their activation on contact with the injured endothelium.

**Fig. 1:**
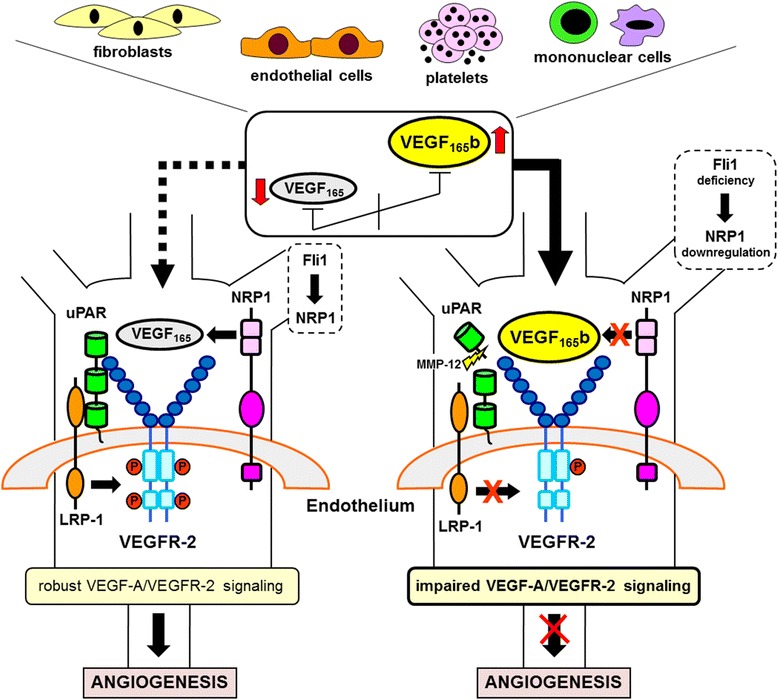
Schematic illustration of the mechanisms impairing VEGF-A/VEGFR-2 angiogenic signal in SSc. Angiogenesis tightly depends on the balance of proangiogenic VEGF_165_ and antiangiogenic VEGF_165_b isoforms. In angiogenic conditions, VEGF_165_ predominates, whereas in SSc the balance shifts to favor the expression of the VEGF_165_b splice variant in different cell types, such as ECs, platelets, fibroblasts, and mononuclear inflammatory cells. In ECs, binding of VEGF_165_ to both VEGFR-2 and NRP1 coreceptor activates VEGFR-2 phosphorylation and multiple downstream signaling pathways that are necessary for angiogenesis. Moreover, uPAR interacts with VEGFR-2 and recruits LRP-1 to VEGFR-2, which induces internalization of VEGF_165_-bound VEGFR-2, a key step in the proangiogenic signal. In SSc, both a switch from proangiogenic VEGF_165_ to antiangiogenic VEGF_165_b, which is unable to bind NRP1, and concomitant NRP1 downregulation due to Fli1 transcription factor deficiency result in an insufficient tyrosine phosphorylation/activation of VEGFR-2 and incomplete or transient downstream signaling along with a differential intracellular trafficking of VEGFR-2 toward the degradative pathway, ultimately leading to impaired angiogenesis. In SSc ECs, MMP-12-mediated cleavage/inactivation of uPAR may further impair the VEGF-A/VEGFR-2 system. *EC* endothelial cell, *Fli1* Friend leukemia integration 1, *LRP-1* low-density lipoprotein receptor-related protein 1, *MMP-12* matrix metalloproteinase-12, *NRP1* neuropilin-1, *SSC* systemic sclerosis, *uPAR* urokinase-type plasminogen activator receptor, *VEGF* vascular endothelial growth factor, *VEGFR* vascular endothelial growth factor receptor

The discovery of VEGF_165_b clearly adds a new fundamental figure in the complex crossroad of the mechanisms underlying dysfunctional angiogenesis in SSc. Binding of VEGF_165_ to vascular endothelial growth factor receptor (VEGFR)-2 induces VEGFR-2 internalization and activates signaling pathways that trigger EC proliferation, survival, and migration which are necessary for angiogenesis. VEGF_165_b binds to VEGFR-2 with the same affinity as VEGF_165_, but binding of VEGF_165_b results in an insufficient tyrosine phosphorylation/activation of VEGFR-2 and incomplete downstream signaling (Fig. 1) [4]. Owing to increased levels of VEGF_165_b, SSc-MVECs show reduced VEGFR-2 phosphorylation and impaired capillary morphogenesis in vitro [5]. Accordingly, the angiogenic performance of SSc-MVECs can be improved by cotreatment with recombinant VEGF_165_ and anti-VEGF_165_b neutralizing antibodies [5]. Of note in the study by Hirigoyen et al. [1], when healthy dermal MVECs were incubated with SSc platelet releasates containing elevated levels of VEGF_165_b, capillary-like tube formation was significantly inhibited compared with cells treated with control platelet releasates.

It is known that the nontyrosine kinase receptor neuropilin-1 (NRP1) acts as a VEGFR-2 coreceptor in angiogenesis [7]. VEGF_165_b antiangiogenic function is thought to be mechanistically related to the lack of NRP1 coreceptor binding [4]. In fact, upon binding of VEGF_165_b to VEGFR-2, the absence of NRP1 cosignaling induces differential trafficking of VEGFR-2 toward proteasomal degradation [4]. In SSc, besides VEGF_165_b overexpression, concomitant NRP1 downregulation because of Friend leukemia integration 1 (Fli1) transcription factor deficiency may further disturb VEGF-A/VEGFR-2 signaling [8]. Finally, in this complex scenario the urokinase-type plasminogen activator receptor (uPAR), an angiogenic orchestrator acting via both VEGF-A-dependent and VEGF-A-independent mechanisms, may play a significant role because uPAR inactivation represents a master factor challenging angiogenesis in SSc [2, 9]. uPAR–VEGFR-2 interaction is crucial for VEGFR-2 internalization and proangiogenic signaling in ECs [10]. Of note, VEGF-A-induced angiogenesis is prevented in uPAR-deficient mice, a recently proposed animal model of SSc [9, 10]. uPAR inactivation/deficiency may thus contribute substantially to VEGF-A/VEGFR-2 system abnormalities in SSc (Fig. 1).

Taken together, considerable evidence suggests that uncontrolled expression of the antiangiogenic VEGF_165_b isoform may take center stage in this crowded crossroad to angiogenesis in SSc. It remains to be elucidated whether the targeting of VEGF_165_b and/or VEGF-A pre-mRNA splicing machinery might represent a promising therapeutic strategy to promote effective angiogenesis in SSc patients. As pointed out in the article by Hirigoyen et al. [1], a better understanding of how platelets transport and deliver uncontrolled levels of VEGF_165_b to the injured endothelium might disclose a novel pathway contributing to the SSc-related peripheral vasculopathy. Whether this mechanism is a priority pathway worth targeting warrants further studies.

## Abbreviations

EC: endothelial cell
Fli1: friend leukemia integration 1
MVEC: microvascular endothelial cell
NRP1: neuropilin-1
SSc: systemic sclerosis
uPAR: urokinase-type plasminogen activator receptor
VEGF: vascular endothelial growth factor
VEGFR: vascular endothelial growth factor receptor
